# From regular text to artistic writing and artworks: Fourier statistics of images with low and high aesthetic appeal

**DOI:** 10.3389/fnhum.2013.00106

**Published:** 2013-04-01

**Authors:** Tamara Melmer, Seyed A. Amirshahi, Michael Koch, Joachim Denzler, Christoph Redies

**Affiliations:** ^1^Experimental Aesthetics Group, Institute of Anatomy I, University of Jena School of Medicine, Jena University HospitalJena, Germany; ^2^Computer Vision Group, Department of Computer Science, Friedrich Schiller UniversityJena, Germany

**Keywords:** writing systems, calligraphy, ornamental art, abstract artworks, spatial frequency, scale invariance, experimental aesthetics

## Abstract

The spatial characteristics of letters and their influence on readability and letter identification have been intensely studied during the last decades. There have been few studies, however, on statistical image properties that reflect more global aspects of text, for example properties that may relate to its aesthetic appeal. It has been shown that natural scenes and a large variety of visual artworks possess a scale-invariant Fourier power spectrum that falls off linearly with increasing frequency in log-log plots. We asked whether images of text share this property. As expected, the Fourier spectrum of images of regular typed or handwritten text is highly anisotropic, i.e., the spectral image properties in vertical, horizontal, and oblique orientations differ. Moreover, the spatial frequency spectra of text images are not scale-invariant in any direction. The decline is shallower in the low-frequency part of the spectrum for text than for aesthetic artworks, whereas, in the high-frequency part, it is steeper. These results indicate that, in general, images of regular text contain less global structure (low spatial frequencies) relative to fine detail (high spatial frequencies) than images of aesthetics artworks. Moreover, we studied images of text with artistic claim (ornate print and calligraphy) and ornamental art. For some measures, these images assume average values intermediate between regular text and aesthetic artworks. Finally, to answer the question of whether the statistical properties measured by us are universal amongst humans or are subject to intercultural differences, we compared images from three different cultural backgrounds (Western, East Asian, and Arabic). Results for different categories (regular text, aesthetic writing, ornamental art, and fine art) were similar across cultures.

## Introduction

During the last decades, the spatial characteristics of letters and their influence on readability and letter identification have been studied in considerable detail (Pelli et al., 2006; Chung and Tjan, 2007; Tyler and Likova, 2007; Chung and Tjan, 2009). A particular focus has been on the spatial frequency components and spacing requirements that facilitate letter identification and improve readability and reading comfort (Solomon and Pelli, 1994; Majaj et al., 2002; Wilkins et al., 2007; Nandy and Tjan, 2008; Oruc and Landy, 2009; Jainta et al., 2010). The statistical image properties that relate to more global aspects of text images, for example properties that may relate to aesthetic aspects of writing systems, have received less attention (for an example, see Wilkins, 1995). Readability and aesthetics are two independent aspects of writing. Artistic writing with intricate ornaments or deformed letters may be highly aesthetic but it can sometimes be difficult to read, for example, Chinese cursive script. In contrast, ordinary (non-artistic) typographic writing is easy to read but may not necessarily be aesthetic.

It has recently been shown that a large variety of aesthetic and other visually pleasing images, including art images of Western and Eastern provenance (Graham and Field, 2007; Redies et al., 2007b; Graham and Redies, 2010) and graphic novels (Koch et al., 2010), exhibit specific statistical properties in their Fourier spectra: with increasing frequency, radially averaged (1d) power falls off according to a power law with a slope of around −2 in log-log plots, i.e., the power spectrum displays 1/*f*^2^ characteristics. This property implies that subsets of visually pleasing images possess a scale-invariant structure in the Fourier domain. Aesthetic images share this property with complex natural scenes (Burton and Moorhead, 1987; Field, 1987; Tolhurst et al., 1992). Vice versa, images that deviate from natural scene statistics can induce visually discomfort (Fernandez and Wilkins, 2008; Juricevic et al., 2010; O'Hare and Hibbard, 2011). Because the mammalian visual system is adapted to process natural scenes with an efficient and sparse sensory code (Olshausen and Field, 1996; Parraga et al., 2000; Vinje and Gallant, 2000; Hoyer and Hyvärinen, 2002; Simoncelli, 2003), it has been proposed that artists create aesthetic images by adapting their artworks to this type of sensory coding in the human visual system (Redies, 2007; Redies et al., 2007a; Graham and Redies, 2010).

It remains unclear, however, whether other types of visual patterns that are produced by humans for viewing in everyday life, also possess 1/*f*^2^ characteristics. Examples are visual patterns that are created without obvious aesthetic intent, such as regular text. In the present study, we therefore compared the statistical properties of regular text with a set of monochrome graphic art analyzed previously (Redies et al., 2007b). Although regular text is not necessarily aesthetic, it may also be adapted or optimized to particular aspects of visual perception, as suggested previously (Wilkins, 1995; Changizi and Shimojo, 2005; Changizi et al., 2006; Jainta et al., 2010). It is therefore of interest to study the statistical properties of text images, not only with respect to local properties, such as the readability of individual letters and words (see above), but also in terms of the global appearance of text images, as suggested previously in a preliminary study on the Fourier spectrum of two examples of Japanese calligraphy and regular print (Ozawa, 1994).

The action of reading is likely to differ from viewing artworks because reading typographic text encompasses the deciphering of a linear code with semantic content (for example, reading line by line from left to right, or top to bottom). In contrast, viewing artworks is much less constrained and the composition of artworks allows the free exploration of global image structure, as shown by eye tracking studies (Wooding et al., 2002; Quiroga and Pedreira, 2011). The differences in viewing strategies likely correspond to differences in statistical image properties. For example, subsets of aesthetic art images were shown to have Fourier spectral properties that are rather uniformly distributed across image orientations (i.e., low anisotropy; Koch et al., 2010). In contrast, images of text are highly anisotropic due to the oriented structure of the lines of text. Moreover, the spatial frequency amplitude that corresponds to the distance between text lines can be expected to be high compared to other frequencies. In the present study of the Fourier spectra of text images, we therefore studied cardinal (horizontal and vertical) and oblique orientations separately to obtain 1d spectra, rather than radially averaging across all orientations together, as usually done when analyzing natural scenes and aesthetic images (see above).

To more closely define the differences in statistical properties between text images and aesthetic art images, we included image categories at the transition between regular text and aesthetic art. Specifically, we studied (1) images of artistic or aesthetic writing (ornamental writing, calligraphy), and (2) aesthetic images that are similar to text images in that they are composed of multiple, largely independent pictorial elements placed side-by-side (ornamental art and abstract expressionist art).

Note that in current experimental research on aesthetics, there is no universally accepted or independent measure for the degree to which an image is aesthetic or artistic. By using these terms for different categories of images in the present study, we rely on views that are commonly held by the general public. Our classifications may well be in conflict with the opinions of individual persons. For example, on the one hand, typographers might claim that the creation of typographic letters is a highly artistic endeavor whereas most of the general public will not regard images of regular printed text as artworks. On the other hand, individual viewers may consider highly acclaimed artworks, such as the drip paintings by Jackson Pollock, unaesthetic. This terminological uncertainty is reflected in the many views on art and aesthetics that abound in philosophy and art history. Consequently, the usage of these terms in the present study should be treated with caution.

Moreover, in view of the great variety of writing styles in different cultures, it was unclear whether any of the statistical properties measured in the present study are universal amongst humans (Changizi and Shimojo, 2005) or are subject to intercultural differences. We therefore compared images from three different cultural backgrounds (Western, East Asian, and Arabic).

Results from the present study contribute to the knowledge on the relationship between the statistical properties of text images and their perceptual processing by the human visual system. By comparing text images to other types of images produced by humans, the present findings contribute also to our understanding of what makes text images special to the human brain.

## Materials and methods

### Image data

The data analyzed in this study include image databases of regular and ornate text of diverse cultures of writing, calligraphy, artistic ornaments of three cultural backgrounds (Western, Arabic, and East Asian), and East Asian and Western fine art (Table 1). A total of 1611 images were analyzed.

**Table 1:** **Slope values for art images and text images**.

	***n***	**5–40 cycles per image**		**40–256 cycles per image**		**Slope difference**
		**Mean slope**	**Mean sigma**	**Mean slope**	**Mean sigma**
**REGULAR PRINT**						
**Latin serif**	77					
Horizontal		0.19 ± 0.20	0.062 ± 0.017	−3.62 ± 0.08	0.061 ± 0.015	3.81 ± 0.58
Oblique		−0.49 ± 0.28	0.061 ± 0.029	−3.44 ± 0.27	0.029 ± 0.011	2.95 ± 0.49
Vertical		−0.68 ± 0.57	0.182 ± 0.170	−2.91 ± 0.25	0.061 ± 0.028	2.23 ± 0.65
**Latin sans serif**	60					
Horizontal		0.09 ± 0.17	0.073 ± 0.016	−3.52 ± 0.04	0.093 ± 0.011	3.61 ± 0.74
Oblique		−0.52 ± 0.27	0.058 ± 0.022	−3.71 ± 0.11	0.045 ± 0.013	3.18 ± 0.62
Vertical		−0.63 ± 0.64	0.166 ± 0.163	−3.43 ± 0.13	0.070 ± 0.021	2.80 ± 0.63
**International serif**	119					
Horizontal		−0.31 ± 0.23	0.067 ± 0.016	−3.49 ± 0.07	0.064 ± 0.009	3.10 ± 0.65
Oblique		−0.78 ± 0.29	0.059 ± 0.023	−3.47 ± 0.19	0.036 ± 0.010	2.65 ± 0.61
Vertical		−0.71 ± 0.32	0.152 ± 0.127	−3.34 ± 0.20	0.063 ± 0.022	2.59 ± 0.68
**HANDWRITING**						
**Carolingian**	36					
Horizontal		−0.04 ± 0.19	0.058 ± 0.015	−3.72 ± 0.08	0.028 ± 0.005	3.69 ± 0.37
Oblique		−0.73 ± 0.27	0.051 ± 0.023	−3.41 ± 0.19	0.022 ± 0.006	2.67 ± 0.33
Vertical		−0.90 ± 0.30	0.187 ± 0.161	−3.87 ± 0.15	0.023 ± 0.013	2.97 ± 0.33
**Latin handwriting**	35					
Horizontal		−1.03 ± 0.21	0.041 ± 0.013	−3.48 ± 0.15	0.010 ± 0.001	2.45 ± 0.41
Oblique		−1.15 ± 0.32	0.045 ± 0.020	−3.45 ± 0.23	0.012 ± 0.005	2.30 ± 0.28
Vertical		−1.02 ± 0.33	0.129 ± 0.065	−3.53 ± 0.12	0.012 ± 0.004	2.51 ± 0.46
**ORNATE PRINT**						
**Latin ornate print**	132					
Horizontal		−0.67 ± 0.27	0.052 ± 0.017	−3.09 ± 0.16	0.035 ± 0.011	2.41 ± 0.71
Oblique		−0.81 ± 0.61	0.053 ± 0.022	−2.75 ± 0.35	0.028 ± 0.011	1.94 ± 0.67
Vertical		−0.82 ± 0.37	0.178 ± 0.141	−2.62 ± 0.33	0.034 ± 0.014	1.80 ± 0.74
**Chinese ornate print**	13					
Horizontal		−0.68 ± 0.35	0.090 ± 0.028	−3.21 ± 0.05	0.052 ± 0.005	2.53 ± 0.45
Oblique		−1.15 ± 0.20	0.080 ± 0.029	−2.94 ± 0.14	0.025 ± 0.006	1.79 ± 0.31
Vertical		−1.16 ± 0.21	0.147 ± 0.138	−2.69 ± 0.17	0.045 ± 0.007	1.53 ± 0.54
**Arabic ornate print**	80					
Horizontal		−0.79 ± 0.42	0.036 ± 0.011	−3.55 ± 0.06	0.113 ± 0.010	2.75 ± 0.28
Oblique		−1.06 ± 0.24	0.053 ± 0.024	−3.54 ± 0.12	0.044 ± 0.016	2.48 ± 0.16
Vertical		−0.44 ± 0.27	0.166 ± 0.107	−3.56 ± 0.08	0.147 ± 0.045	3.12 ± 0.32
**CALLIGRAPHY**						
**Arabic cursive**	39					
Horizontal		−0.91 ± 0.47	0.043 ± 0.017	−3.29 ± 0.14	0.043 ± 0.017	2.38 ± 0.92
Oblique		−0.86 ± 0.25	0.048 ± 0.021	−3.17 ± 0.17	0.030 ± 0.011	2.31 ± 0.81
Vertical		−0.88 ± 0.43	0.109 ± 0.059	−3.51 ± 0.15	0.035 ± 0.014	2.63 ± 0.90
**Chinese cursive**	92					
Horizontal		−0.99 ± 0.28	0.117 ± 0.031	−3.52 ±0.06	0.034 ± 0.007	2.54 ± 0.63
Oblique		−0.95 ± 0.29	0.057 ± 0.027	−3.27 ± 0.13	0.023 ± 0.009	2.31 ± 0.43
Vertical		−1.25 ± 0.53	0.050 ± 0.016	−3.42 ± 0.12	0.034 ± 0.011	2.17 ± 0.53
**ORNAMENTS**						
**Grotesque ornaments**	69					
Horizontal		−1.08 ± 0.24	0.066 ± 0.017	−2.65 ± 0.06	0.016 ± 0.002	1.58 ± 0.55
Oblique		−1.02 ± 0.25	0.051 ± 0.020	−2.64 ± 0.07	0.013 ± 0.002	1.62 ± 0.43
Vertical		−1.27 ± 0.64	0.056 ± 0.021	−2.62 ± 0.06	0.016 ± 0.004	1.34 ± 0.51
**Arabic ornaments**	253					
Horizontal		−0.90 ± 0.31	0.069 ± 0.019	−2.89 ± 0.09	0.017 ± 0.004	2.04 ± 0.86
Oblique		−0.79 ± 0.30	0.070 ± 0.026	−2.79 ± 0.12	0.015 ± 0.004	2.03 ± 0.76
Vertical		−0.94 ± 0.46	0.066 ± 0.022	−2.83 ± 0.11	0.017 ± 0.005	1.94 ± 0.74
**East Asian ornaments**	78					
Horizontal		−2.10 ± 0.28	0.052 ± 0.020	−3.62 ± 0.13	0.003 ± 0.002	1.55 ± 0.70
Oblique		−2.02 ± 0.24	0.053 ± 0.020	−3.96 ± 0.26	0.018 ± 0.006	1.94 ± 0.81
Vertical		−1.97 ± 0.22	0.053 ± 0.015	−3.76 ± 0.11	0.012 ± 0.003	1.78 ± 0.82
**FINE ART**						
**Abstract expressionism**	119					
Horizontal		−1.80 ± 0.24	0.047 ± 0.02	−2.66 ± 0.08	0.028 ± 0.01	0.86 ± 0.65
Oblique		−1.76 ± 0.31	0.046 ± 0.02	−2.51 ± 0.11	0.019 ± 0.00	0.74 ± 0.72
Vertical		−1.84 ± 0.29	0.045 ± 0.02	−2.67 ± 0.08	0.024 ± 0.00	0.82 ± 0.64
**European fine art**	200					
Horizontal		−2.13 ± 0.24	0.045 ± 0.01	−1.95 ± 0.09	0.011 ± 0.00	−0.18 ± 0.81
Oblique		−2.20 ± 0.30	0.044 ± 0.02	−1.86 ± 0.15	0.009 ± 0.00	−0.34 ± 0.81
Vertical		−2.15 ± 0.28	0.043 ± 0.02	−1.89 ± 0.12	0.043 ± 0.02	−0.25 ± 0.73
**East Asian fine art**	209					
Horizontal		−2.05 ± 0.25	0.046 ± 0.01	−2.34 ± 0.08	0.012 ± 0.00	0.19 ± 0.70
Oblique		−2.13 ± 0.27	0.044 ± 0.02	−2.20 ± 0.14	0.011 ± 0.00	0.07 ± 0.68
Vertical		−2.13 ± 0.26	0.042 ± 0.01	−2.26 ± 0.09	0.012 ± 0.00	0.14 ± 0.73

#### Regular print, handwriting, ornate print, and calligraphy

For the analysis of text images, we scanned the largest possible square section comprising eight lines of monochrome original text. Care was taken to select original print samples reproduced at a high quality and at a size that was sufficiently large. Scanning was performed at a high resolution (400 dpi) in 8-bit gray scale with a scanner (Perfection 3200 Photo, Epson, Nagano, Japan) that was calibrated as described previously (Redies et al., 2007a). Subsequently, resizing each image to 1024 × 1024 pixels reduced the resolution. For each category of text, different original documents were used. The number of sections taken from one original document ranged from about 1 to 4.

For Latin serif and sans serif fonts, a sample text was set in 77 serif fonts and in 60 sans serif fonts with the Photoshop program (Adobe, Mountain View, CA). For international serif fonts, 119 examples of the same text from different writing systems were generated with the Photoshop program, including samples from Europe (Latin, Georgian, Kyrillic, Greek), the Middle East (Hebrew, Arabic), North America (Cherokee), North India (Devanagari, Gujarati, Nagari, Oriya), South India (Sinhala, Tamil, Telugu), South East Asia (Laotian, Khmer, Thai), Africa (Ethiopian), and the Far East (Chinese, Japanese, Korean). By the same method, examples of ornate print (117 Latin samples, 13 Chinese samples, 80 Arabic samples) were generated.

To assess differences within one font, we generated 30 samples each of different text passages that were set in Times New Roman font (Latin serif), Arial font (Latin sans serif), and a Georgian font (serif font), respectively. Moreover, we analyzed digital images directly for analysis without printing and scanning. As a control, we compared the digital images with the same images printed on paper and scanned as described above. The differences between the different types of images were small (data not shown).

For Latin and Carolingian handwriting, examples were scanned from two books on these subjects (Menz, 1912; Klemm, 1998). We also gathered 39 different examples of Arabic calligraphy by scanning three different books on the subject. The background of the images was rendered white by subtracting it in the Photoshop program. As a control, we also analyzed the original scans of the same images. Results reveal differences in the low-frequency range, most likely caused by the paper structure. However, these differences have only a minor influence on the slopes (data not shown).

From reproductions in various textbooks on East Asian calligraphy, 92 examples of Chinese calligraphy representing different styles and periods were scanned. Some of the images showing eight lines of text were not square due to a smaller size of the original artworks in the other direction (fewer columns of text). Before their reduction to 1024 × 1024 pixels, the images were padded according to square ones with the MatLab program by adding a uniform border with a gray level that was equal to the mean gray level of the scanned image, as described previously (Redies et al., 2007a).

#### Ornaments

For Western grotesque ornaments, 69 different samples were scanned from a textbook (Warncke, 1979), as described above. With a digital camera (Canon, Ixus 400), 78 photographs of East Asian ornaments were taken from samples of 17th and 18th century Chinese and Japanese porcelain (vases, pots, and dishes) that were on display at the Dresden Porcelain Collection in Dresden, Germany. Square details from the objects were analyzed (for an example, see Figure 13A). Photographs of Arabic ornaments (253 images) were taken with a digital camera (Canon EOS 500D) from interior and exterior wall reliefs of the Alhambra Palace complex in Grenada, Spain. The palace represents an example of 14th century Moorish architecture (for an example, see Figure 12A).

#### Western and east asian fine art and abstract expressionism

For Western art, a previously analyzed dataset of 200 examples of monochrome graphic art of Western provenance was used (Redies et al., 2007b). For East Asian (China, Japan, and Korea) fine art, 209 images of monochrome paintings were collected. Both datasets were scanned from diverse high-quality art books on the subject, as described above, and represented a large variety of graphic styles, subject matters, techniques, centuries, and artists. Largest possible square details from the artworks were analyzed. One-hundred nineteen examples of monochrome Abstract Expressionist art by four artists (32 paintings by Jackson Pollock, 18 paintings by Jean Dubuffet, 59 paintings by Cy Twombly, and 10 paintings by Christian Dotremont) were scanned from artbooks. Images were padded according to square ones, as described above. To render the images more similar to those of printed text, the background was subtracted from the images. The calculated slopes did not differ substantially between the original scanned images and the images after background subtraction (data not shown). As another control, we compared largest possible details of the East Asian art images to padded versions of the same images. Again, differences were small (data not shown).

### Image analysis

#### Radial averaging of fourier power

**Figure 1:**
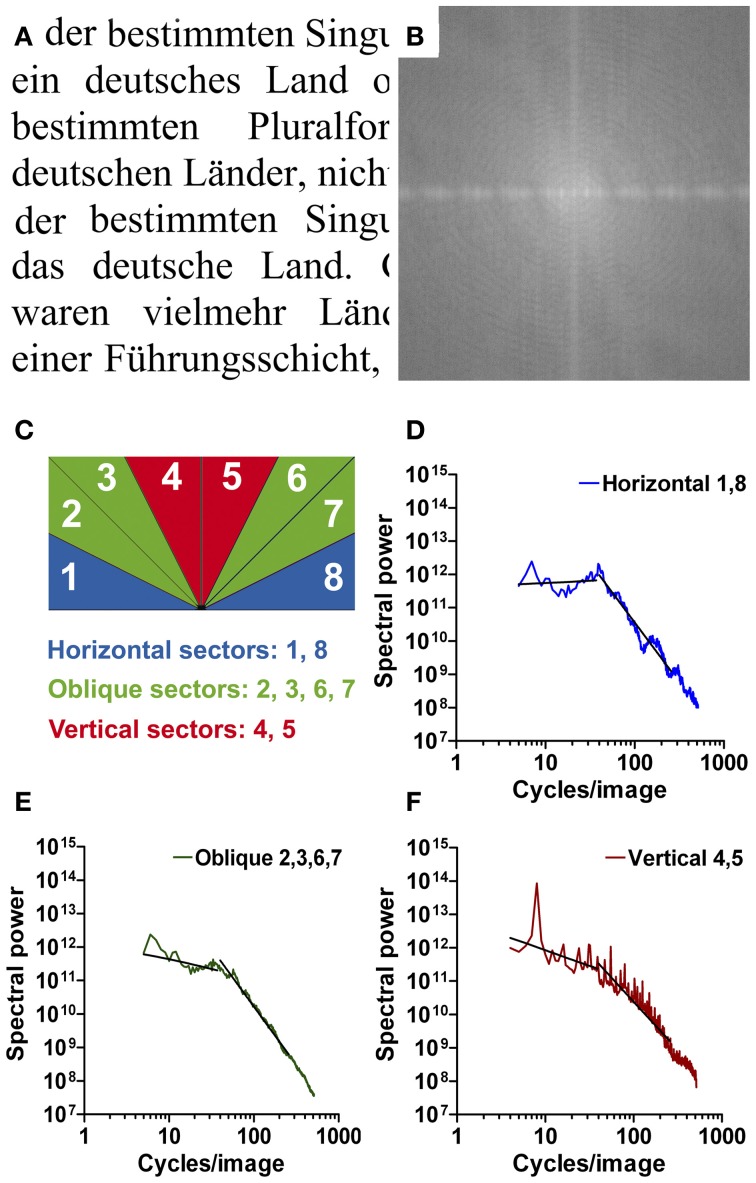
**An example of Latin printed text (Times New Roman font; A) with its 2d Fourier power spectrum (B).** The sectors that were used to calculate Fourier power for horizontal (blue, **D**), oblique (green, **E**), and vertical orientations (red, **F**) are schematically shown in **(C)**. Radially averaged Fourier power is plotted as a function of spatial frequency in the log-log plane in **(D–F)**. The two straight lines in each plot were fitted to the curve in the frequency ranges of 5–40 cycles/image and 40–256 cycles/image, respectively.

Image analysis was performed using Matlab. If required, images were resized to a resolution of 1024 × 1024 pixels by bicubic interpolation using the *imresize* function in Matlab. For each image, the power spectrum (amplitude squared) was obtained by using an efficient algorithm for computing the discrete Fourier transform (2d Fast Fourier Transform). The 2d Fourier power spectrum of each image (for example, see Figure 1B) was divided into eight equal sectors (Figure 1C). For each sector, the 2d spectrum was transformed to a 1d power spectrum by rotational averaging for each frequency (Redies et al., 2007b). Values were combined for (near-)horizontal orientations (sector 1 [0–22.5°] and sector 8 [157.5–180°]; blue in Figure 1C), (near-)vertical orientations (sectors 4, 5 [67.5–112.5°]; red in Figure 1C), and oblique orientations (sectors 2, 3 [22.5–67.5°] and sectors 6, 7 [112.5–157.5°]; green in Figure 1C). Power was then plotted for horizontal orientations (Figure 1D), oblique orientations (Figure 1E), and vertical orientations (Figure 1F) as a function of spatial frequency in the log-log plane.

For regular print, the resulting plots consisted of two roughly linear parts: a low-frequency part (range 5–40 cycles per image, cpi) with a shallow slope and a high-frequency part (range 40–256 cpi) with a steeper slope. This finding was similar for horizontal, oblique, and vertical orientations. As expected with images of eight horizontal lines of text, a prominent peak at 8 cpi was observed for vertical orientations (Figure 1F).

#### Slope of 1d fourier plots

To measure the slopes of the curves in the two parts of the frequency spectrum, data points were binned at regular frequency intervals in the log-log plane and a least-squares fit of a line was performed separately for each of the two parts of the spectrum, as described previously (Redies et al., 2007b). Compared to the fitting to one continuous second-order polynomial function with three parameters, the fitting of two separate lines (with two intercepts and two slopes as parameters) allowed us to relate our present results more directly to previous slope measurements (Burton and Moorhead, 1987; Tolhurst et al., 1992; Graham and Field, 2007; Redies et al., 2007a,b; Koch et al., 2010).

For the high-frequency part, fitting was restricted to frequencies up to 256 cpi to minimize artifacts due to rectangular sampling and raster screen. For the low-frequency part, fitting was restricted to frequencies down to 5 cpi to avoid absent sample points for some orientations and to exclude information that is not of interest (artifacts due to uneven illumination and mean gray level). Moreover, for vertical orientations, values corresponding to the peak at around 8 cpi (7–9 cpi) were not included in the line fitting. As a measure of the goodness of the fit, we determined the mean deviation of the data points from the fitted lines (sigma in Table 1). Table 1 lists the values as means for each image category [±1 standard deviation (SD)].

Because a characteristic difference between images of artworks and text seemed to be the change in the log-log plots of radially averaged Fourier power at around 40 cpi (see Results), we also calculated the difference between the slopes of the low-frequency and high-frequency parts.

#### Anisotropy

As outlined in the Introduction section, the Fourier power spectrum of text images is likely to differ across orientations (anisotropy) because of the horizontal text lines (or vertical text lines in the case of Chinese writing). To analyze this anisotropy, we determined the average absolute difference between the power values for horizontal orientations (sectors 1 and 8 in Figure 1C) and vertical orientations (sectors 4 and 5 in Figure 1C) for each image. To calculate the difference, data were sampled at equal frequency intervals in the log-log plots of Fourier power for each image (see, e.g., Figures 1D–F). Differences were normalized to the mean power for vertical and horizontal orientations for each data point.

## Results

**Figure 2:**
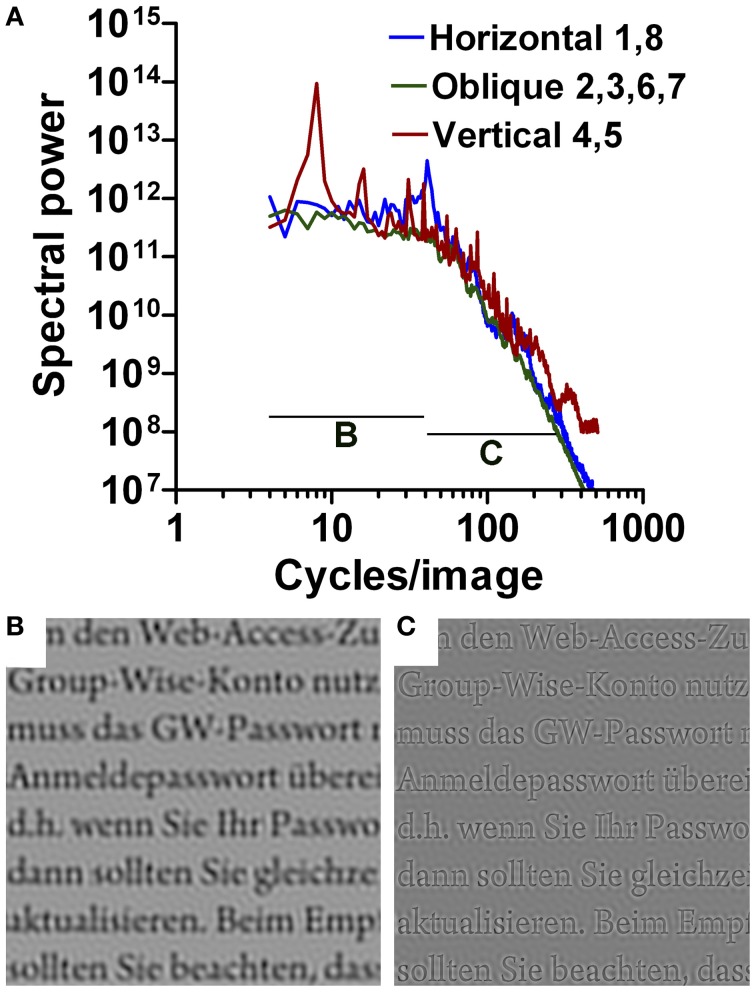
**Results for Latin printed text (Arno Pro font).** The plot in **(A)** shows an overlay of the plots for horizontal, oblique, and vertical orientations, as indicated (see Figure 1C). Band pass-filtered images are displayed for 5–40 cpi in **(B)**, and for 40–256 cpi in **(C)**.

Figures 1, 2 illustrate the type of Fourier analysis performed in the present study. As an example, the results for images of regular print (Times New Roman serif font, Figure 1; Arno Pro serif font, Figure 2) are shown. In the Fourier power spectrum (Figure 1B), low spatial frequencies are represented at the center and high frequencies at the periphery. Lighter shades represent more spectral power. For each frequency, power was radially averaged in sectors representing cardinal (horizontal and vertical) and oblique orientations (Figure 1C) and plotted as a function of spatial frequency in separate log-log plots (Figures 1D–F).

In contrast to similar plots for images of natural scenes or artworks (Burton and Moorhead, 1987; Field, 1987; Tolhurst et al., 1992; Graham and Field, 2007; Redies et al., 2007a,b), the plots for regular text can be roughly divided into two parts (Figure 2A). In the low-frequency part of the spectrum, the average curves for the cardinal and oblique orientations are more shallow; the plot for vertical orientations (red curve in Figure 2A) contains a major peak at about 8 cpi that corresponds to the periodicity of text lines in the images, as expected. To visualize this low-frequency part of the spectrum for the reader, a bandpass-filtered (5–40 cpi) representation is displayed in Figure 2B; it shows a blurred version of the text image. In the high-frequency part, the curves are steeper and fall-off linearly in the log-log plots. In the bandpass-filtered representation of the high-frequency part (40–256 cpi; Figure 2C), the outlines of all letters are sharply demarcated. The two parts of the curves form a transition at around 40 cpi for images with eight lines of text. In control images with four lines and 16 lines of Latin printed text, the transition is shifted to about 20 and 80 cpi, respectively (data not shown), suggesting that the transition point is found at a spatial frequency about five times higher than the frequency peak that reflects the number of lines.

### Anisotropy

**Figure 3:**
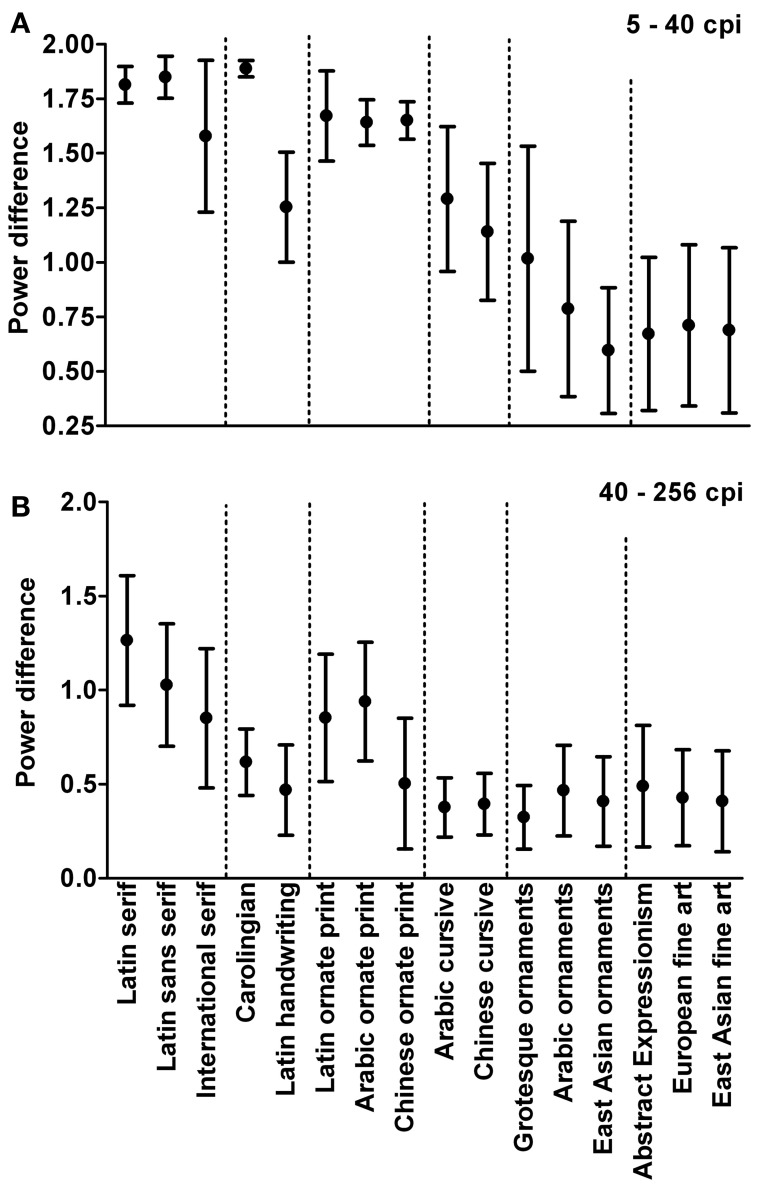
**Average power difference between horizontal and vertical orientations (anisotropy) for different image categories.** Anisotropy values are given for the low-frequency part (5–40 cpi; **A**) and the high-frequency part (40–256 cpi; **B**) of the Fourier spectrum. Image categories are arranged roughly according to artistic claim, which increases from left to right. Values represent the mean ± 1 SD.

Figure 3 shows the mean difference between power values for vertical and horizontal orientations (anisotropy) for all image categories. Results are arranged with subjective artistic claim increasing from left to right, from regular print and handwriting to ornate print, ornamental art, calligraphy, and artworks. As a word of caution, however, we note that a concept like artistic claim is difficult to quantify and may be subject to various philosophical and art historical considerations (see Introduction). Results are presented separately for the low-frequency part (Figure 3A) and the high-frequency part of the spectrum (Figure 3B).

As expected, anisotropy values are high for regular print in both parts of the spectrum. Similarly high values are obtained for ornate print and for Carolingian handwriting that resembles regular print in its uniform stroke width and regular letter alignment. Values are lower for fine art (*p* < 0.001), confirming previous results (Koch et al., 2010), and for East Asian ornaments (porcelain decorations) and Arabic ornaments (wall decorations) (*p* < 0.001). Significances of the differences between the different image categories were determined by the Tukey range test throughout this work. For the low-frequency part of the spectrum (Figure 3A), values for Latin handwriting, calligraphy, and grotesque ornaments are intermediate between artworks and regular print (*p* < 0.001). For the high-frequency part (Figure 3B), these image categories are about as lower or higher than those of fine art.

### Slope measurements

To quantify the steepness of the curve in the low-frequency part (5–40 cpi) and in the high-frequency part (40–256 cpi), we calculated the slopes of straight lines that were fitted to the curves in the two ranges for each image (for examples, see Figures 1D–F). We also measured the differences between the slopes of the high-frequency and low-frequency parts (see Materials and Methods section). For a continuous straight line through both ranges, this difference assumes a value of 0. For each category of images, the two slopes and their difference are listed in Table 1 for the two cardinal (vertical and horizontal) and the oblique orientations. Moreover, to assess how well these straight lines fitted the curves, the deviation (sigma) of the curves from the fitted lines was also determined. In the following sections, we will describe the results for each of the image categories that were analyzed in the present study.

#### General overview

**Figure 4:**
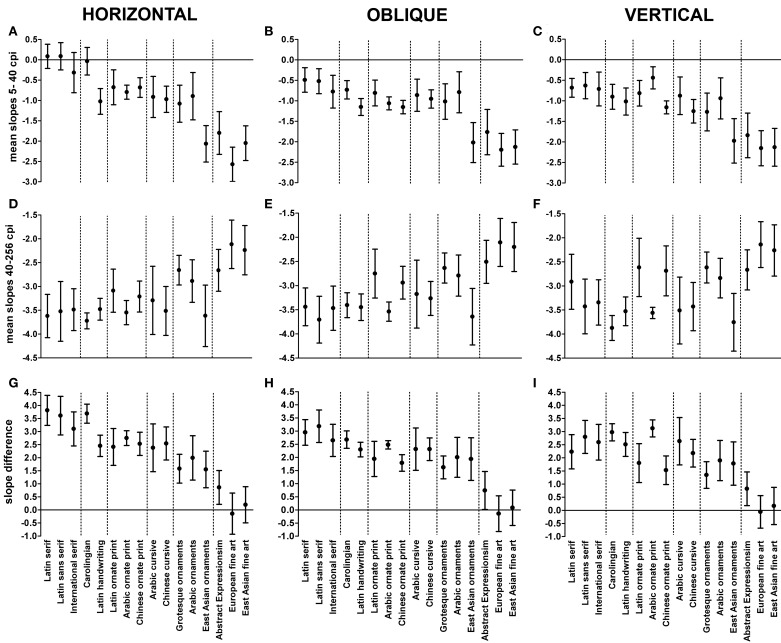
**Average slope values for different image categories.** Mean slope values are plotted for the low-frequency part (5–40 cycles/image; **A–C**) and the high-frequency part (40–256 cycles/image; **D–F**) of the Fourier spectrum. The average differences of the slope values are shown in **(G–I)**. Image categories are arranged roughly according to artistic claim, which increases from left to right. Results for horizontal orientations are displayed in **(A,D,G)**, for oblique orientations in **(B,E,H)** and for vertical orientations in **(C,F,I)**. Values represent the mean ± 1 SD.

Like in Figure 3, results are arranged with artistic claim increasing from left to right in Figure 4. In this direction, the slope in the low-frequency part (5–40 cpi; Figures 4A–C) becomes more negative and approaches values between around −2 and −2.5. The slope in the high-frequency part assumes less negative values and approaches similar slope values (40–256 cpi; Figures 4D–F). As a result, the difference between the two slopes decreases with increasing artistic claim (Figures 4G–I) and approaches values of about 0. These general tendencies are similar for all three orientations ranges.

#### Regular print

**Figure 5:**
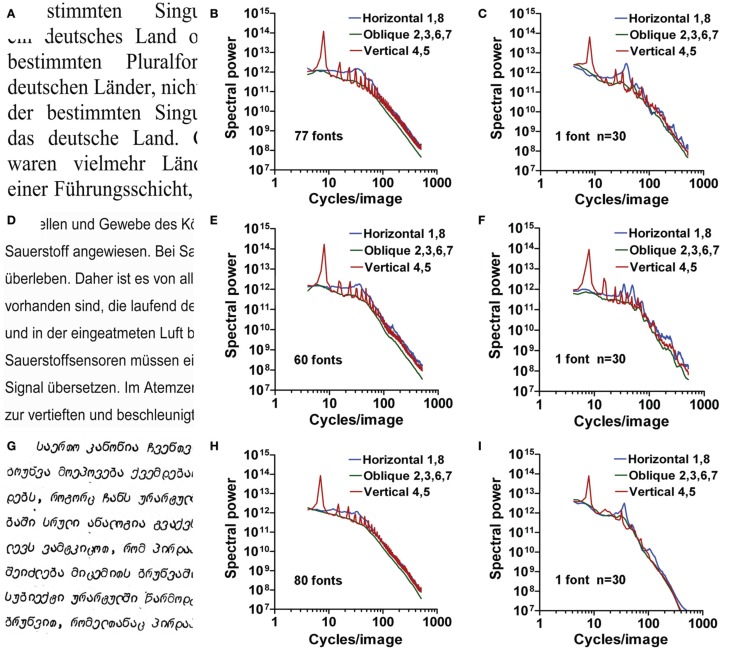
**Results for images of regular print.** Results for Latin serif fonts **(A–C)**, Latin sans serif fonts **(D–F)**, and international fonts **(G–I)** are shown. Exemplary images are displayed in the left column **(A,D,G)**. Radially averaged Fourier power is plotted as a function of spatial frequency in the log-log plane in **(B,C,E,F,H,I)**. The middle column **(B,E,H)** displays mean results for one sample of multiple fonts. The right column **(C,F,I)** displays mean results for 30 samples of a single font. The colors of the plots represent the different orientations (see Figure 1C).

To study whether different categories of regular printed text result in similar graphs, we carried out the same type of analysis for multiple Latin fonts (serif and sans serif) as well as examples of regular print of other provenances (Arabic, Chinese, and other international fonts). An example of each type of font is displayed in Figures 5A,D,G. Averaged curves for all fonts within one category (one example for each font) are shown in Figures 5B,E,H and averaged curves for 30 examples of one font are displayed in Figures 5C,F,I. The within-font variance was similar or less than the between-font variance (data not shown).

For all plots in Figure 5, the steepness of the curve changes at around 40 cpi, similar to the example shown in Figure 1. The mean slopes for the low-frequency part (−0.78 to 0.19) indicate that power is constant or falls less strongly with increasing frequency than for the high-frequency part (mean slopes between −3.71 and −2.91). The slope difference assumes mean values between 2.23 and 3.81 (Table 1; Figure 4).

#### Artworks

**Figure 6:**
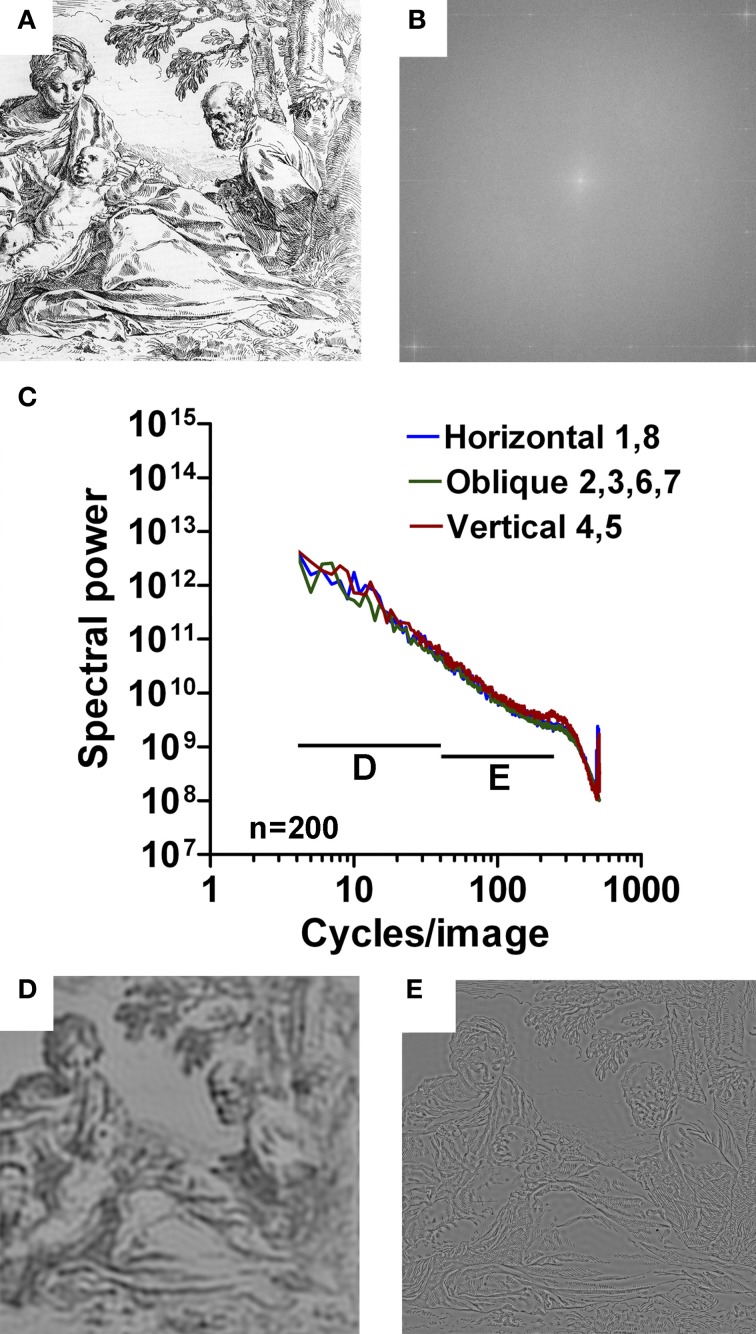
**Results for images of Western graphic art.** As an example, an etching by Simone Cantarini (about 1640; **A**) is shown with its 2d Fourier spectrum **(B)**. Mean radially averaged Fourier power for the 200 images of Western graphic art (Redies et al., 2007b) is plotted as a function of spatial frequency in the log-log plane in **(C)**. The colors of the plots represent the different orientations (see Figure 1C). Band pass-filtered images of the image in **(A)** are shown for 5–40 cpi in **(D)**, and for 40–526 cpi in **(E)**.

**Figure 7:**
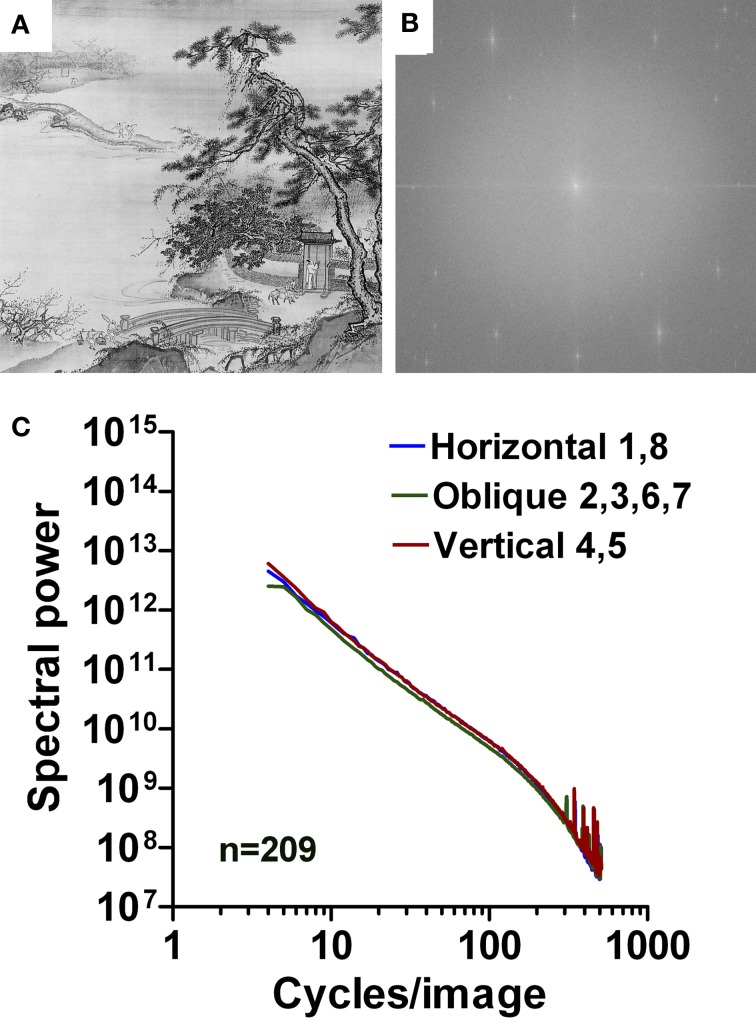
**Results for images of East Asian paintings.** As an example, a detail of a hanging scroll by Tai Chin, a 15th century Chinese artist, is shown in **(A)** with its 2d Fourier spectrum **(B)**. Mean radially averaged Fourier power for the 209 images of East Asian paintings is plotted as a function of spatial frequency in the log-log plane in **(C)**. The colors of the plots represent the different orientations (see Figure 1C).

To quantify the expected difference between regular print and artworks, we carried out the same type of analysis for two different datasets of artwork images, namely 200 examples of European graphic fine art (Redies et al., 2007b; Graham and Field, 2008), and 209 examples of East Asian monochrome paintings and prints. Figures 6A, 7A show examples of the original images analyzed, together with their 2d Fourier power spectra (Figures 6B, 7B). With increasing spatial frequency, power falls nearly linearly according to a power law (1/*f*^2^ characteristics) for all orientations (Figures 6C, 7C). The band-pass filtered images that correspond to the image in Figure 6A are shown in Figures 6D,E. Confirming previous studies (Redies et al., 2007b; Graham and Field, 2008), there is no abrupt transition at 40 cpi between the slopes of the low-frequency part and the high-frequency part.

The results listed in Table 1 confirm that the slopes of art images is more negative (*p* < 0.001; Tukey range test) than images of regular print and assume average values around −2 for the low-frequency part of the spectrum (range −1.76 to −2.20). In the high-frequency part, the slope is less negative (*p* < 0.001) with average values between −1.86 and −2.67, depending on the orientations. The slope difference is around 0 (i.e., close to a straight line; range −0.34 to 0.86) and differs from printed text (*p* < 0.001; Table 1, Figures 1–5).

#### Handwriting

**Figure 8:**
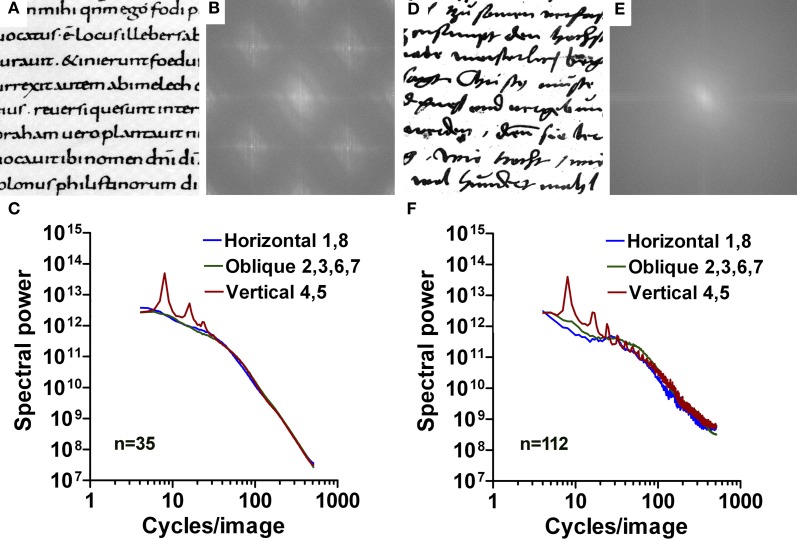
**Results for images of handwritten text. (A–C)** Carolingian handwriting. **(D–F)** Latin handwriting from the Reformation age. Exemplary images are shown in **(A,D)** with their respective 2d Fourier power spectrum in **(B,E)**. Mean radially averaged Fourier power for the 36 samples of Carolingian writing and the 35 samples of Latin handwriting is plotted as a function of spatial frequency in the log-log plane in **(C)** and **(F)**, respectively. The colors of the plots represent the different orientations (see Figure 1C).

Next, we asked whether samples of handwritten text share some of the features observed for regular print. We analyzed two types of historical Latin handwriting. Figure 8 show results from a Carolingian manuscript (10th century; Figures 8A–C) and for handwritten Latin text from the Reformation period (dated 1528; Figures 8D–F), respectively. For Carolingian writing, overall results are similar to those of regular print. Similar to regular print, Carolingian handwriting is characterized by a uniform stroke width and a regular vertical alignment and spacing of the letters. For Latin handwriting, slopes are more negative in the low-frequency part (range −1.02 to −1.15; *p* < 0.001) and the slope difference is smaller for horizontal and oblique orientations than for regular print (2.45 and 2.30, respectively; *p* < 0.001; Table 1; Figure 4).

#### Ornate print and calligraphy

**Figure 9:**
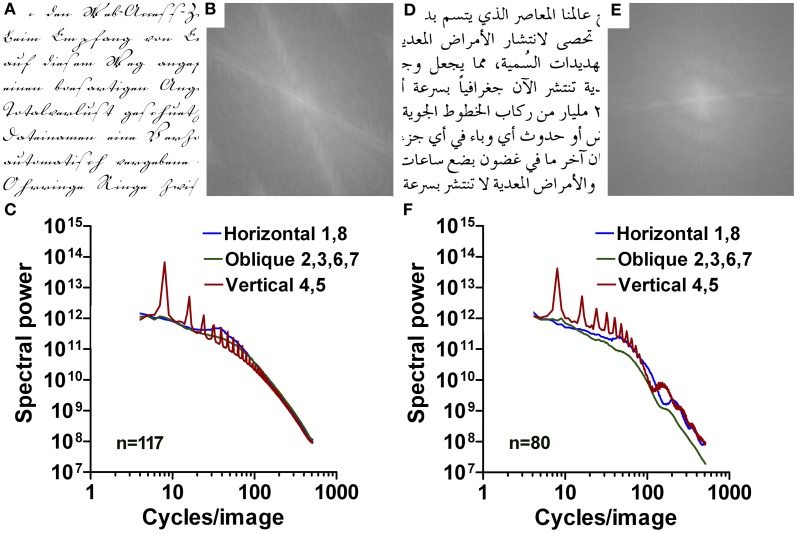
**Results for images of Latin (A–C) and Arabic ornate print (D–F).** Exemplary images are shown in **(A,D)** with their respective 2d Fourier power spectrum in **(B,E)**. Mean radially averaged Fourier power for the 132 samples of Latin ornate print and the 80 samples of Arabic ornate print is plotted as a function of spatial frequency in the log-log plane in **(C)** and **(F)**, respectively. The colors of the plots represent the different orientations (see Figure 1C).

**Figure 10:**
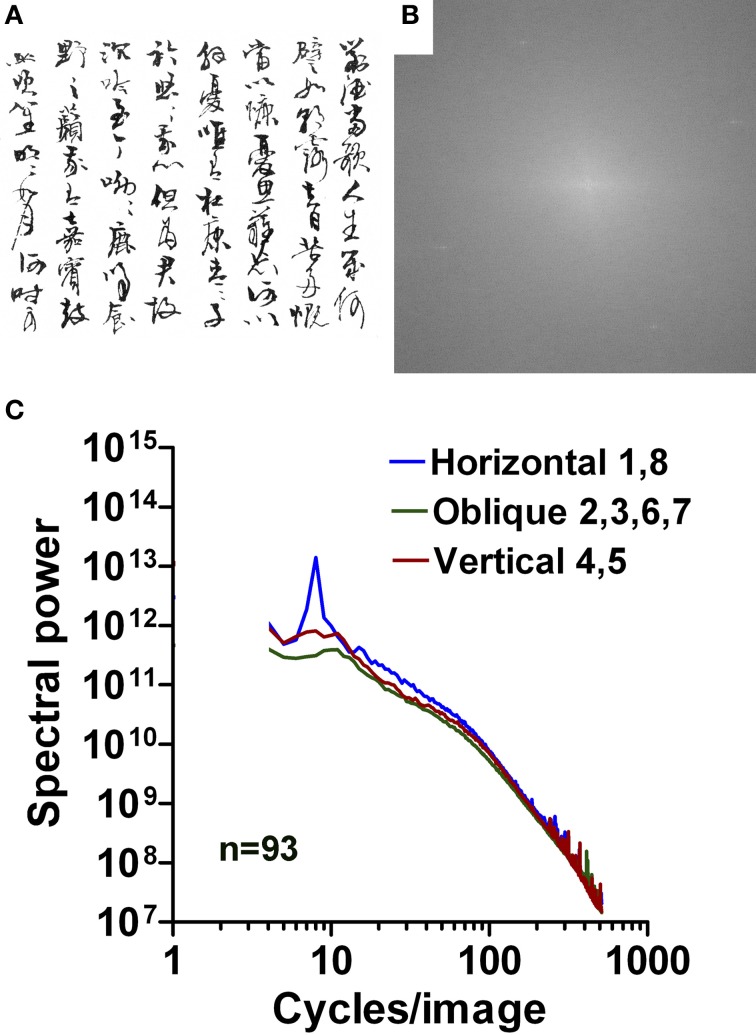
**Results for images of Chinese calligraphy.** An example is shown in **(A)** with its 2d Fourier spectrum in **(B)**. Mean radially averaged Fourier power for the 92 samples of Chinese cursive script is plotted as a function of spatial frequency in the log-log plane in **(C)**. The colors of the plots represent the different orientations (see Figure 1C).

We also analyzed writing systems with artistic claim. Firstly, we analyzed ornate fonts of three different cultural provenances (Latin, Arabic, and Chinese). Results for Latin and Arabic ornate print are shown in Figure 9. Secondly, we analyzed Arabic and Chinese cursive calligraphy. Typically, the cursive Arabic letters have long ascenders and descenders, while cursive Chinese pictograms are characterized by quick, fluent brushwork. Figure 10 shows results for Chinese calligraphy.

Similar to the samples of Latin handwriting, slope values for the horizontal direction (range −0.67 to −0.99) and vertical direction (range −0.44 to −1.25) are smaller than for regular print (*p* < 0.001) and higher than those for aesthetic artworks (*p* < 0.001) in the low-frequency part of the spectrum. An exception is Arabic ornate print with values that are similar to regular print for the slopes of the high-frequency part and the slope difference. Results for the other orientations and parts of the spectrum are similar to those of regular print.

#### Ornamental art

**Figure 11:**
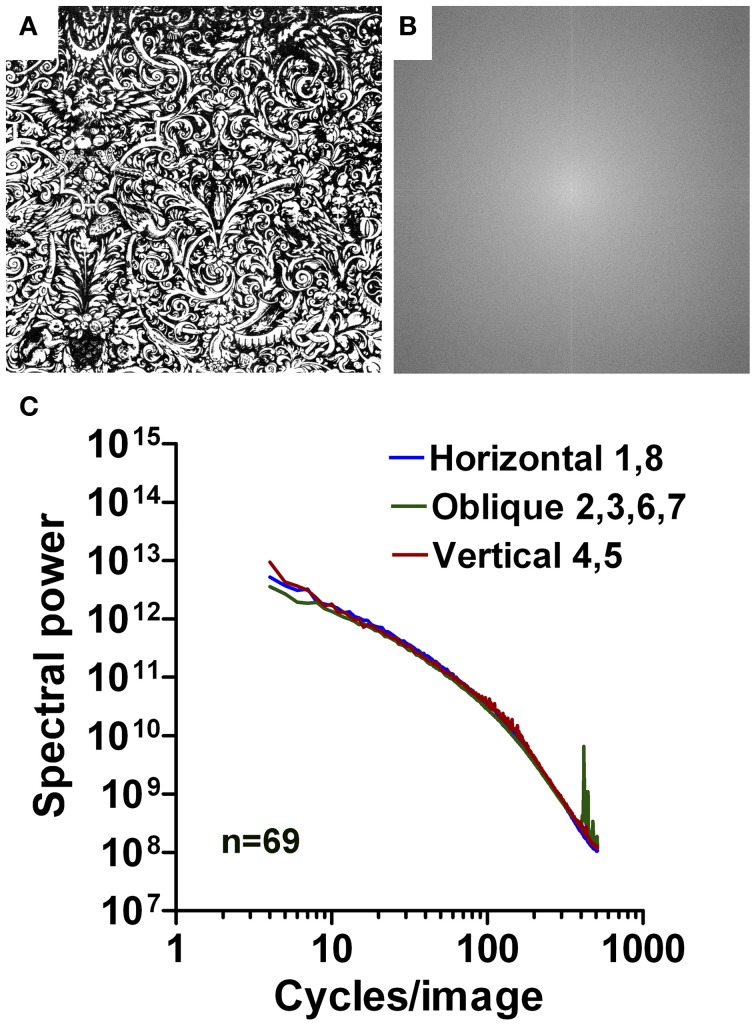
**Results for images of Western grotesque ornaments.** An example is shown in **(A)** with its 2d Fourier spectrum in **(B)**. Mean radially averaged Fourier power for the 69 samples is plotted as a function of spatial frequency in the log-log plane in **(C)**. The colors of the plots represent the different orientations (see Figure 1C).

**Figure 12:**
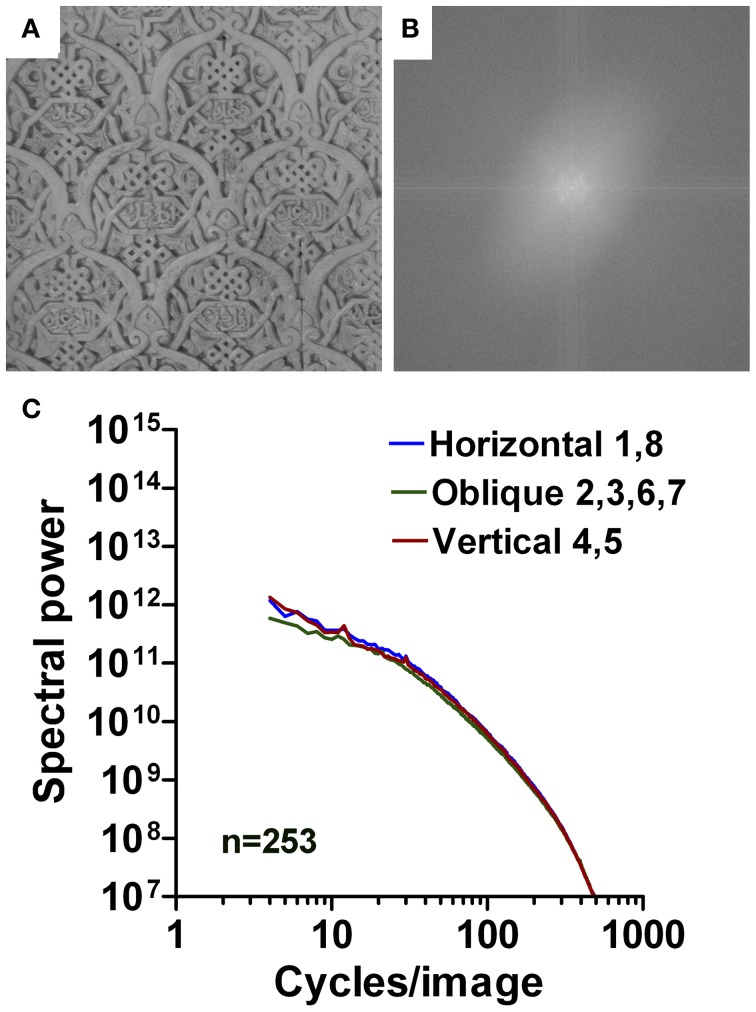
**Results for images of Arabic ornaments (wall reliefs).** An example is shown in **(A)** with its 2d Fourier spectrum in **(B)**. Mean radially averaged Fourier power for the 253 samples is plotted as a function of spatial frequency in the log-log plane in **(C)**. The colors of the plots represent the different orientations (see Figure 1C).

**Figure 13:**
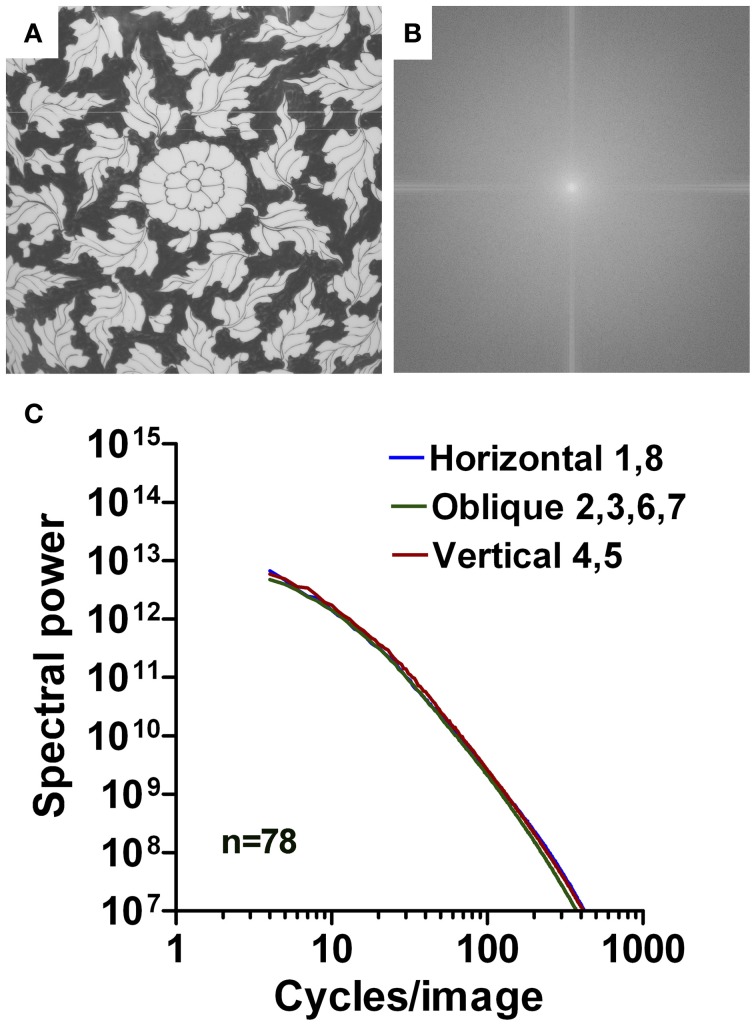
**Results for images of East Asian ornaments (porcelain decorations).** An example is shown in **(A)** with its 2d Fourier spectrum in **(B)**. Mean radially averaged Fourier power for the 78 samples is plotted as a function of spatial frequency in the log-log plane in **(C)**. The colors of the plots represent the different orientations (see Figure 1C).

The arrangement of letters in regular text without artistic claim can be described as a sequence of largely independent pictorial elements. In contrast, artworks are characterized by a more global composition, in which individual pictorial elements relate to each other throughout the entire image (see Introduction). We next studied images of ornamental art (Western, Arabic, and East Asian), which consist also of repetitive pictorial elements arranged in a global structure (Figures 11–13).

The mean log-log plots for the three datasets are characterized by a more gradual rather than an abrupt transition of Fourier power at around 40 cpi (Figures 11C, 12C, 13C). Results for Western grotesque ornaments (Figure 11) and ornamental Arabic art (wall decorations; Figure 12) are similar to those of ornate print and calligraphy in general. For the low-frequency part of the spectrum, the slope values for ornamental paintings on East Asian porcelain (Figure 13) are lower than for Western and Arabic ornamentals (*p* < 0.001) and resemble those of aesthetic artworks (Figures 4A–C). The opposite tendency is observed for the high-frequency part of the spectrum (Figures 4D–F; *p* < 0.001). Here, slope values for East Asian decorative art are similar to those of regular print and lower than those of Western and Arabic decorative art (*p* < 0.001). For all orientation ranges, slope differences (Figures 4G–I) are equal or higher (*p* < 0.001) for ornamental art than for images of ornamental writing or calligraphy.

#### Abstract expressionism

Finally, particular styles of abstract art can be described as an arrangement of similar pictorial elements embedded in a global image structure, similar to ornamental art. In the present work, we analyzed paintings by Abstract Expressionist artists (Jackson Pollock, Jean Dubuffet, Cy Twombly, and Christian Dotremont). Examples of the images cannot be shown for copyright reasons.

The mean log-log plot for the Abstract Expressionist dataset (not shown) is similar to the curves for fine art. The slope values in the low-frequency range are around −1.8 (Table 1; Figures 4A–C), which is only slightly lower that the value for European and Asian fine art and East Asian porcelain decorations (around −2.1, *p* < 0.001), but lower (*p* < 0.001) than the mean value for artistic Western and Arabic ornaments (around −1.0). In the high-frequency part (Figures 4D–F), however, the curve for abstract expressionism assumes slope values of around −2.6. This is lower (*p* < 0.001) than the value for fine art (−1.9). The slope differences (Figures 4G–I) are around 0.74–0.86, compared to 1.34–2.04 for artistic ornaments (*p* < 0.001) and −0.34 −0.19 for fine art (*p* < 0.001).

## Discussion

In the present work, we compared image statistics of ordinary text and different categories of images with artistic claim. Humans create all these images for viewing by humans. As a consequence, the images may exhibit statistical properties that reflect sensory adaption to the human visual system (Changizi et al., 2006; Graham and Redies, 2010). However, the aesthetic appeal and artistic intent of the image categories differ (see Introduction). Our results reveal that, in general, specific statistical properties vary with the artistic claim of the images.

### Images of regular print are not scale-invariant

It can be expected that aesthetic artworks and regular text differ in their Fourier power spectra (see Introduction). In the present work, we provide a systematic study of this assumption and quantify the differences by applying a computer-based algorithm for measuring statistical image properties. For aesthetic artworks, radially averaged spectral power falls off roughly linearly according to a power law (1/*f*^*p*^ characteristics) with increasing spatial frequency in log-log plots; the mean slope value [*p*] is about −2 (Graham and Field, 2007; Redies et al., 2007a,b; Graham and Redies, 2010). This result implies that the Fourier spectrum is scale-invariant. Artworks share this property with other types of aesthetically pleasing images (for example, graphic novels and comics; Koch et al., 2010) and with images of complex natural scenes (see Introduction). Fractal-like structure was also found in particular types of music (Voss and Clarke, 1975; Beauvois, 2007), architecture (Joye, 2007), and American sign language (Bosworth et al., 2006). Unlike artworks, regular print has a steeper slope in the high-frequency part of the power spectrum (value of about −3.5) while the slope of the low-frequency part is shallower (value of about −1.2, Table 1, Figure 4). The difference in the slope values between the three orientation ranges (horizontal, oblique, and vertical) is small. Similar findings for regular handwritten text suggest that, in images of regular text, power in the low-frequency part of the spectrum is relatively low when compared to artworks, with the exception of a prominent peak at 8 cpi (see above). Because low spatial frequencies represent coarse structure in an image and high spatial frequencies represent fine detail, this result implies that images of ordinary text tend to contain a lower amount of global image structure than the artworks analyzed. In the artworks, the higher amount of global structure may represent a physical correlate of artistic composition, which relates individual pictorial elements to each other across the image (see Introduction).

It has been argued that all images produced by the human hand, including artworks, generally possess scale-invariant properties for reasons related to the nature of hand movements (e.g., see Graham and Field, 2008). The present findings indicate that not all hand-made images are scale-invariant in the Fourier domain. The result that images of handwriting contain a lower amount of global image structure is not surprising because global image structure is not important for text, which is scanned word-by-word in a consecutive manner. In addition, even the high-frequency part of the Fourier power spectra of text is not scale-invariant. We conclude that humans can manufacture images that may or may not be scale-invariant, depending on their purpose.

Studies on artificial images revealed that manipulations of the Fourier spectrum can elicit visual discomfort if a significant deviation from scale invariance is induced (Fernandez and Wilkins, 2008; Juricevic et al., 2010; O'Hare and Hibbard, 2011). In particular, images with a curvilinear amplitude spectrum and an energy upshot at about 3–4 cycles per degree, i.e., close to the position where the visual system is most sensitive, can elicit visual discomfort (Fernandez and Wilkins, 2008). Visual discomfort is often (but not always) negatively associated with artistic merit (Fernandez and Wilkins, 2008). In the present study, curvilinear power spectra are also observed for several of the image categories (for example, images of text, calligraphy, and ornamental art), but the images used by us do not evoke obvious visual discomfort in general. Consequently, it remains unclear how the findings by Fernandez and Wilkins (2008) relate to our present results.

Together, these results suggest that several types of images, which lack scale invariance, are of relatively low aesthetic value. The opposite notion, however, does not hold because images, which possess scale-invariant Fourier spectra, are not necessarily aesthetic (for example, some computer-generated images; Lee and Mumford, 1999). It has therefore been suggested that scale invariance is a corollary of some other (unknown) feature of aesthetic images that contributes to aesthetic perception (Redies et al., 2007a).

### Slope difference and anisotropy correlate with artistic claim

Images of text are of particular interest for studying aesthetic perception because there is a continuous transition from regular print to aesthetic writing (ornamental writing and calligraphy) and to visual art (see Introduction). Our results (Table 1, Figures 3, 4) illustrate that, with increasing artistic claim, images of text acquire specific statistical properties that are similar to those of visual art. As one such measure, we introduced the difference of the slopes between the low- and high-frequency parts of the radially averaged power spectrum. With increasing artistic claim, this difference decreases to values close to 0 (straight line) for all orientation ranges (Table 1, Figure 4), which indicates a nearly linear fall-off throughout the entire frequency spectrum, similar to what has been observed for natural scenes (1/*f*^2^ characteristics). A similar transition between non-aesthetic images and images with artistic claim is observed for the horizontal/vertical power difference (anisotropy measure; Figure 3). For images of text, differences between vertical and horizontal orientations probably reflect the periodicity of the text lines and/or regularities in the width or spacing of the lines that form the letters. Compared to regular text, the Fourier spectrum of fine art represents all orientations at similar strength, as shown previously by Koch et al. (2010) who compared artworks to other categories of images. This result is not trivial because artists could easily produce paintings, in which particular orientations predominate. In how far low anisotropy is necessary or sufficient to induce aesthetic perception—and in which types of fine art—remains to be studied.

In our analysis, we included a special style of art (Abstract Expressionism) that resembles text images in its repetitive arrangement of multiple and simple pictorial elements distributed over a large surface area. Nevertheless, the statistical properties of Abstract Expressionist paintings are more similar to those of other art images than to ordinary text, suggesting that they contain a high amount of global structure, similar to fine art. This result is also not trivial because, conceivably, paintings using the same pictorial elements could be produced with a lower amount of global structure. Our results are compatible with the suggestion by R. Taylor and other researchers that abstract expressionist paintings, like the drip paintings by Jackson Pollock, possess fractal-like structure (Taylor, 2002; Mureika, 2005; Alvarez-Ramirez et al., 2008).

Other examples of repetitive structures arranged over a large surface are ornamental decorations, which are also created to be enjoyed by human observers but may perhaps have lesser artistic claim in general. The slope differences for all three types of decorative art are intermediate between those of fine art and ordinary text. The anisotropy values of decorative art are intermediate or closer to fine art, when compared to regular print (Figure 3). Intermediate values are also obtained for calligraphy, a writing style with artistic claim. It remains unclear whether other types of images show a similar relation between statistical regularities and artistic claim.

### Similar findings in text images of different cultural provenance

To compare Western, Arabic, and East Asian examples of the different categories of text images (Table 1, Figures 3, 4), we chose a horizontal approach and compared contemporary text images that include plain and ornate fonts as well as serif and sans serif fonts of different international alphabets. In addition, we chose a vertical approach and analyzed text of different ages (medieval manuscripts, calligraphy, and Reformation handwriting). Overall, we analyzed 11 datasets, each including between 13 and 253 samples (1598 images in total; Table 1).

Results from the Fourier analysis were generally similar for the three cultural backgrounds (Table 1, Figures 3, 4). In particular, the slope differences were similar for Latin and international serif fonts of regular print, and also for ornate print, calligraphy, and ornamental art from all three cultures. Moreover, our results confirm that artworks from both East Asian and Western provenance possess similar scale-invariant properties in the Fourier domain (Redies et al., 2007b; Graham and Field, 2008). Together, these results suggest that specific perceptual mechanisms for reading and aesthetic judgment, respectively, may be common amongst humans across different cultural backgrounds. It has been speculated that such common principles may have emerged due to selective pressures imposed by the adaptation of the human visual system to specific perceptual and motor tasks during the evolution of mankind (Changizi and Shimojo, 2005; Changizi et al., 2006; Redies, 2007; Graham and Redies, 2010).

Last but not least, physical features of the visual inputs have been shown to strongly modulate the functional responses in some core regions of the reading network, including for instance the influence of spatial frequency on the activation of the left ventral occipitotemporal cortex (Seghier and Price, 2011; Woodhead et al., 2011; Horie et al., 2012). The kind of image statistics studied here may thus shed some light on how the human brain processes written word stimuli in comparison to other types of stimuli. This topic warrants future studies.

### Conflict of interest statement

The authors declare that the research was conducted in the absence of any commercial or financial relationships that could be construed as a potential conflict of interest.
